# Chicago Medical Society

**Published:** 1889-05

**Authors:** 


*Special Meeting, February 27.*

Dr. F. E. Waxham presented a patient wearing both an intubation and a tracheotomy tube with very little discomfort, the only annoyance being the necessity for changing the tracheotomy tube once or twice a week.

The case was interesting on many accounts: First, on account of the age of the patient. Laryngeal growths are very uncommon in children, but when met with they are almost always benign and papillomatous in character. Papillomata of the larynx are rarely met with in a child so young as this one, being only 2½ years of age when the first operation was performed for papilloma, now being about 3 years old.

The history of this case was interesting also as showing the great tolerance of the larynx for laryngeal tubes.

Dr. H. Gradle read a paper entitled

REPORT OF A CASE OF TRANSPLANTATION OF THE SKIN FOR THE CURE OF ECTROPIUM, which appeared in the March number of The Journal and Examiner.

Dr. Horace M. Starkey presented

A CASE ILLUSTRATING WOLFE'S METHOD OF SKIN TRANSPORTATION, WITH REMARKS, the patient having received burns on October 27th, 1887, by explosion of a tank of varnish which was being boiled in the process of manufacture. His head, face, neck and hands were badly burned. Over six months from the receipt of the injury elapsed before any attempt was made to lessen, by operative measures, the great deformity that had been occasioned.

May 3d, 1888, an attempt was made to improve the position of the right lower lid as follows: A curved and pointed flap of integument 2.75 millimetres (1⅛ inches) broad at the base and 13 centimetres (5 inches) long on its convex border, was raised from its bed, and while not detached at the base was revolved in such a way as to raise the lid to its proper position. This left an uncovered surface 1.5 centimetres (⅝ inches) broad and 8 centimetres (3 inches) long. To cover this a piece of skin somewhat crescent-shaped 14.5 centimetres (5¾ inches) on its convex border and 2.5 centimetres (1 inch) broad was detached from the inner side of the forearm and placed like a picture in a frame on the denuded surface. Only a part of this transported portion retained its vitality permanently. There was no sloughing, but part became mummified and seemed to dissolve in the second week.

The failure of most of this graft to finally live is perhaps due to a fault in the details of the operation, of which I will speak later. Owing to the failure of part of the graft the improvement which at first was very considerable was not very marked after the lapse of three or four months.

July 21st, 1888, the ectropium, which was not extreme, of the left lower lid was corrected by the operation devised by Wharton Jones.

After seven months from the continued contraction there is now a slight separation of the lid from the eyeball.

The second operation upon the right side was performed October 31st, 1888, and consisted in freeing both eyelids from the cicatricial bands holding them, dissecting out some of the most dense of those bands, restoring as nearly as possible the lids to their normal position, and transporting from the forearm pieces of skin large enough to fill the deficiency. The transported graft for the upper lid was 5 centimetres (2 inches) long by 17.5 millimetres ($\frac{3}{4}$ inch) broad, and that for the lower lid was 7.5 centimetres (3 inches) by 2.5 centimetres (1 inch). These both retained their vitality perfectly and now constitute part of the skin of the corresponding parts and can scarcely be distinguished from the surrounding tissue.

While the improvement from this operation was very marked and has remained permanent for the upper lid, the lower lid was gradually everted until the ectropium amounted to about 12.5 millimetres ($\frac{1}{2}$ inch), and January 7th, 1889, another transportation was performed. In this instance the graft was 5.5 centimetres ($2\frac{1}{4}$ inches) by 2 centimetres ($\frac{7}{8}$ inch). The lid is not in close apposition with the eyeball, and it is doubtful if it can be made to assume that position permanently, but the improvement in appearance and in the comfort of the patient is very great.

